# Auxin influx importers modulate serration along the leaf margin

**DOI:** 10.1111/tpj.12921

**Published:** 2015-07-27

**Authors:** Ania Kasprzewska, Ross Carter, Ranjan Swarup, Malcolm Bennett, Nick Monk, Jamie K. Hobbs, Andrew Fleming

**Affiliations:** ^1^ Department of Animal and Plant ScienceUniversity of SheffieldSheffieldS10 2TNUK; ^2^ Department of Physics & AstronomyUniversity of SheffieldSheffieldS10 2TNUK; ^3^ Centre for Plant Integrative BiologyUniversity of NottinghamSutton BoningtonLE12 5RDUK; ^4^ School of Mathematics and StatisticsUniversity of SheffieldSheffieldS10 2TNUK; ^5^ Present address: Polish Academy of Sciences Botanical GardenCenter for Biological Diversity ConservationWarsawPoland

**Keywords:** auxin, leaf, shape, morphogenesis, *Arabidopsis thaliana*, modelling

## Abstract

Leaf shape in Arabidopsis is modulated by patterning events in the margin that utilize a PIN‐based auxin exporter/CUC2 transcription factor system to define regions of promotion and retardation of growth, leading to morphogenesis. In addition to auxin exporters, leaves also express auxin importers, notably members of the *AUX1*/*LAX* family. In contrast to their established roles in embryogenesis, lateral root and leaf initiation, the function of these transporters in leaf development is poorly understood. We report that three of these genes (*AUX1*,* LAX1* and *LAX2*) show specific and dynamic patterns of expression during early leaf development in Arabidopsis, and that loss of expression of all three genes is required for observation of a phenotype in which morphogenesis (serration) is decreased. We used these expression patterns and mutant phenotypes to develop a margin‐patterning model that incorporates an *AUX1*/*LAX1*/*LAX2* auxin import module that influences the extent of leaf serration. Testing of this model by margin‐localized expression of *axr3–1* (*AXR17*) provides further insight into the role of auxin in leaf morphogenesis.

## Significance Statement

Auxin transport plays a core role in plant development. We show that a well‐characterised family of auxin importers displays complex dynamic patterns of expression during early leaf development which influences the degree of serration, thus adding to our understanding of the role of auxin in leaf morphogenesis.

## Introduction

Research from a number of groups has characterized the key role that the growth regulator auxin plays in both the patterning of organ initiation at the apical meristem (leading to arrangements of leaves around the main stem axis: phyllotaxis) and in subsequent patterning events that occur along the leaf margin (Reinhardt *et al*., 2003; Jönsson *et al*., 2006; de Reuille *et al*., 2006; Smith *et al*., 2006; Barkoulas *et al*., 2008; Bilsborough *et al*., 2011). Central to the various models that have been proposed to interpret these observations is polar auxin transport (PAT), by which spatial and temporal control of PIN‐FORMED (PIN)‐mediated auxin export allows the generation of spatially discrete auxin signaling maxima (Benková *et al*., 2003; Vanneste and Friml, 2009). With respect to patterning along the leaf margin, primordia initially have a smooth perimeter that may become more elaborate over developmental time via the formation of lobes and serrations. In Arabidopsis, the pattern of serration is dictated by the PAT system in conjunction with the CUP‐SHAPED COTYLEDON2 (CUC2) family of transcription factors, with a pattern of alternate auxin‐response maxima and CUC2 maxima forming along the leaf perimeter (Bilsborough *et al*., 2011). The sites of auxin‐response maxima coincide with regions of relative tissue outgrowth, whereas CUC2 maxima coincide with regions of retarded growth, thus leading to a pattern of serrations along the edge of the leaf. In addition, Bilsborough *et al*. (2011) showed that serration patterning may be described by a relatively simple model in which the leaf margin is depicted by a one‐dimensional chain of cells within which the PAT/CUC2 patterning system operates. Using a series of ordinary differential equations to simulate synthesis/breakdown of auxin and CUC2, and implementing rules by which cellular auxin concentration dictates PIN distribution in neighboring cells (the so‐called ‘up the gradient’ rule) and the presence of CUC2 modulates PIN expression, they created a model that generates alternate patterns of auxin‐response maxima and CUC2 maxima similar to those observed in real leaves. However, although they captured an essential element of leaf margin patterning, it did not completely recapitulate all elements of patterning observed in real leaves. For example, in wild‐type (WT) Arabidopsis leaves, serration is restricted to the base of the leaf, whereas the model generated pattern along the entire leaf margin. This discrepancy may be accommodated by assuming differential growth along the leaf proximal/distal axis. However, measurements of areal growth rate in young Arabidopsis leaves do not reveal any major gradients along the proximal/distal axis at the very early stages when serrations normally form, although there is a gradual decrease in areal growth rate in the distal region as the leaf enlarges that may act to decrease serration growth in this part of the leaf (Kuchen *et al*., 2012). These differences between model and observation suggest that some biological elements may be missing from the model that, when implemented, would allow a fuller understanding of how the margin patterning system works.

In the case of auxin, it is well established that other transporters exist in addition to the PIN family of proteins, and particular attention has been paid to the *AUX1*/*LAX* gene family (Bennett *et al*., 1996; Swarup *et al*., 2005; Peret *et al*., 2012; Robert *et al*., 2015). A general conclusion has been that these importers have the potential to modify the outputs of the PAT system. Thus, for example, loss of AUX1/LAX activity leads to altered phyllotaxis, suggesting that these auxin importers function within the meristem to stabilize auxin signalling maxima (Bainbridge *et al*., 2008). Consistent with these observations, modeling approaches have supported the function of auxin importers in stabilizing auxin patterns initiated via PIN‐based transport systems, for example in vascular differentiation (Kramer, 2004; Heisler and Jonsson, 2006). However, although specific *AUX1*/*LAX* genes are expressed in leaf primordia (Bilsborough *et al*., 2011), a role for *AUX1*/*LAX* genes in leaf morphogenesis has not been identified.

In this paper, we describe a series of experiments to investigate the function of *AUX1*/*LAX* genes in the control of leaf shape. We show that three *AUX1*/*LAX* genes (*AUX1*/*LAX1*/*LAX2*) display specific and dynamic patterns of expression during early leaf development in Arabidopsis, and that loss of expression of all three genes is required for observation of a phenotype in which serration is decreased. We incorporate these patterns into a leaf margin patterning model, and show how an *AUX1*/*LAX1*/*LAX2* auxin import module may influence the degree of auxin accumulation and thus serration growth. Finally, we consider the interaction of organ growth and patterning (both of which may be modulated by auxin) and the potential role of auxin sensitivity in the margin as a factor determining the morphogenic outcome of the patterning system.

## Results

### AUX1/LAX genes show dynamic expression patterns during early leaf development

**Figure 1:**
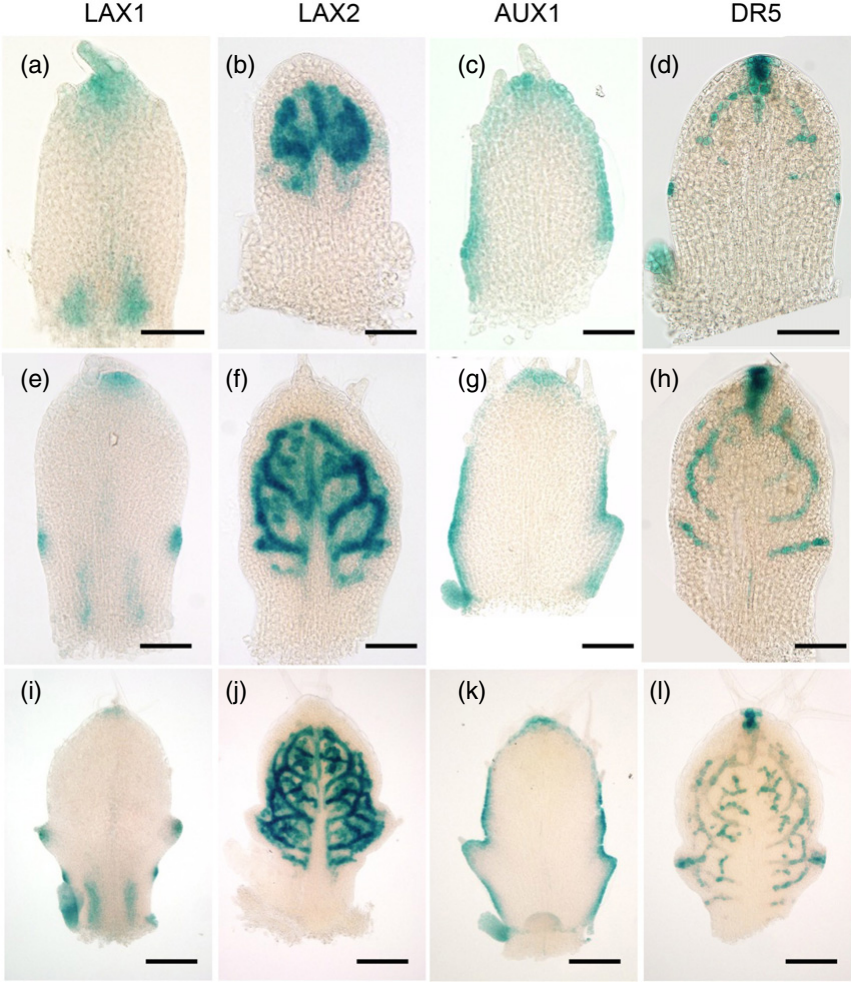
AUX1/LAX genes show dynamic patterns of expression during early leaf development. Expression patterns observed in Pro_LAX__1_:GUS (a,e,i), Pro_LAX__2_:GUS (b,f,j), Pro_AUX__1_:GUS (c,g,k) and Pro_DR__5_:GUS (d,h,l) transgenic lines. Patterns are shown for leaf 5, which was removed at an early stage of development before overt initiation of serration (a–d), at a stage when the first serration is initiated (e–h), and at a later stage when the first serration is clearly formed (i–l). The GUS signal is blue. Scale bars = 50 μm (a–d), 100 μm (e–h) and 200 μm (i–l).

**Figure 2:**
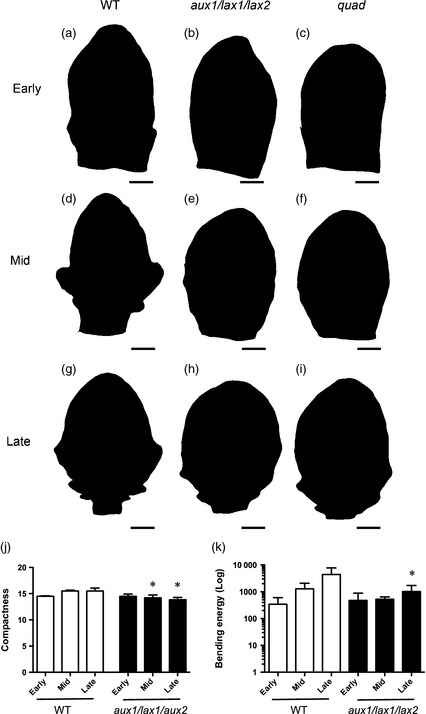
Mutation of three auxin importers is required for altered serration growth. (a–i) Silhouettes of exemplar leaf 5 at an early stage of leaf development when serration is just initiating in WT primordia (a–c), at a mid‐stage of development when the first serration has clearly formed in WT (d–f), and at a late stage of development when multiple serrations have formed (g–i). Leaf shapes are shown for WT (a,d,g), *aux1*/*lax1*/*lax2* (b,e,h) and quadruple (*quad*) mutant plants (c,f,i). Scale bars = 50 μm (a–c), 200 μm (d–f) and 500 μm (g–i). (j,k) Quantitative analysis of compactness (j) and bending energy (k) are shown for early‐, mid‐ and late‐stage leaves dissected from WT and *aux1*/*lax1*/*lax2* plants. ANOVA using a Kruskal–Wallis test indicated that the samples were statistically significantly different for both parameters at *P *<* *0.01 (compactness) and *P *<* *0.05 (bending energy) (*n *=* *6). Subsequent pairwise Mann–Whitney non‐parametric tests for each developmental stage indicated that mid‐ and late‐stage *aux1*/*lax1*/*lax2* samples were statistically significantly different for compactness compared with their WT counterparts at *P *<* *0.05 (indicated by an asterisk), and late‐stage *aux1*/*lax1*/*lax2* samples were statistically significantly different for bending energy compared with their WT counterpart at *P *<* *0.05 (indicated by an asterisk).

Previous analyses have reported that *AUX1*,* LAX1* and *LAX2* are expressed in the shoot apex, whereas *LAX3* is not expressed in aerial tissue (Bainbridge *et al*., 2008). To provide a more detailed analysis of these expression patterns, we used transgenic Arabidopsis Col–0 plants expressing promoter–GUS constructs to document the temporal and spatial pattern of *AUX1*/*LAX* gene expression throughout leaf development. Focusing first on *LAX1* expression (Figure 1a,e,i), the GUS reporter signal was initially apparent in a group of cells at the tip of the leaf. As development proceeded, points of *LAX1* expression were observed along the flanks of the leaf in an approximately symmetrical pattern just proximal to the mid‐point of the proximal/distal axis, indicating the tips of the presumptive serration outgrowth (Figure 1e). At later stages, new points of *LAX1* expression appeared along the leaf margin proximal to the original sites of *LAX1* expression, again indicating presumptive sites of serration (Figure 1i). In addition to expression at points along the margin, signal was also often (but not always) observed towards the base of the leaf in internal positions. With respect to *LAX2* expression (Figure 2b,f,j), GUS activity was initially restricted to internal tissue towards the distal region of the primordium in a complex pattern. As the leaf developed, the network of cells expressing the *LAX2* reporter shifted towards the base of the leaf, with the lower boundary of expression being approximately at a line defined by the most proximal points of *LAX1* expression (Figure 1f). *LAX2* expression was always excluded from the outer cell layers, and gradually became restricted to a network resembling part of the differentiating vascular system (Figure 1j). *LAX3* expression was not detectable in leaf tissue (Bainbridge *et al*., 2008). *AUX1* expression has previously been reported to be restricted to the meristem epidermis and margin of the emerging leaf primordia (Reinhardt *et al*., 2003; Heisler and Jonsson, 2006), and our analysis of an *AUX1* promoter reporter gene construct broadly confirmed this expression pattern throughout the stages of leaf development (Figure 1c,g,k). At the earliest stages of development, some reporter gene expression was observed in the sub‐marginal cells, but we cannot exclude the possibility of some signal diffusion in these small samples. The synthetic DR5 promoter construct has been widely used as a reporter of auxin signaling (Ulmasov *et al*., 1997; Sabatini *et al*., 1999), and analysis of plants transformed with a DR5::GUS construct indicated that, at an early stage of development, signal was apparent at the leaf tip and two equidistant points on the margin, as well as in internal strands in the distal region (Figure 1d) (Mattsson *et al*., 2003). At later stages of development, Pro_DR5_:GUS signal became apparent both at points along the leaf margin, indicative of presumptive outgrowth, and in a network within the leaf reminiscent of regions of presumptive vascular differentiation (Figure 1h,l) (Scarpella *et al*., 2006; Wenzel *et al*., 2007).

### Combined loss of three auxin importer genes leads to a delay in leaf serration

To investigate the effect of loss of *AUX1*/*LAX* gene function on leaf shape, we analysed a series of leaves at various developmental stages from a range of single, double, triple and quadruple *aux1*/*lax* mutants (Bainbridge *et al*., 2008; Peret *et al*., 2012). A phenotype was only observed in the triple mutant *aux1*/*lax1*/*lax2* and the quadruple (*quad*) mutant in which all four *aux1*/*lax* genes were mutated (Figure 2). In WT leaves, the early primordium had a relatively smooth margin with slight undulations towards the base (Figure 2a). Subsequently, overt serration occurred at positions towards the leaf base (Figure 2d). As development proceeded, serrations arose in more proximal positions (Figure 2g); however, the higher relative growth rate of the main body of the leaf compared to the serrations meant that these outgrowths were not as pronounced in the mature leaf as in earlier stages of leaf development, as previously described (Kuwabara *et al*., 2011). In the *aux1*/*lax1*/*lax2* mutants, serrations were not visible at early leaf stages (Figure 2b,e) but were apparent during later stages (Figure 2h). These serrations formed in an appropriate position (i.e. towards the base of the leaf), but differential growth was limited so the serrations were never as pronounced as those observed in WT leaves. Similarly, *quad* mutants showed a lack of overt serration during early primordium development (Figure 2c,f) but serrations were apparent at later stages (Figure 2i), although again these were never as pronounced as in the WT and the overall leaf shape tended not to be as symmetrical as in WT. In contrast, the leaf margins of *aux1*/*lax1*,* aux1/lax3* and *aux1*/l*ax2* double mutants mutants were indistinguishable from those of the WT at all developmental stages (Figure S1). The growth rate of the *aux1*/*lax1*/*lax2* and *quad* leaves was slower than for WT or single or double mutant combinations. To allow a quantitative comparison of leaf shape during development in the various genetic backgrounds, we used LEAFPROCESSOR software (Backhaus *et al*., 2010) to analyse leaf profiles normalized for size differences. Our previous work had indicated that two parameters are especially useful for discriminating 2D leaf shape in different genetic backgrounds: compactness (a measure of the ratio of circumference to area) and bending energy (a measure of integrated curvature around the leaf perimeter). As shown in Figure 2(j,k), the late stage leaves of *aux1*/*lax1*/*lax2* plants had statistically significantly lower values for these parameters compared with WT leaves, consistent with a smoother, less serrated shape.

**Figure 3:**
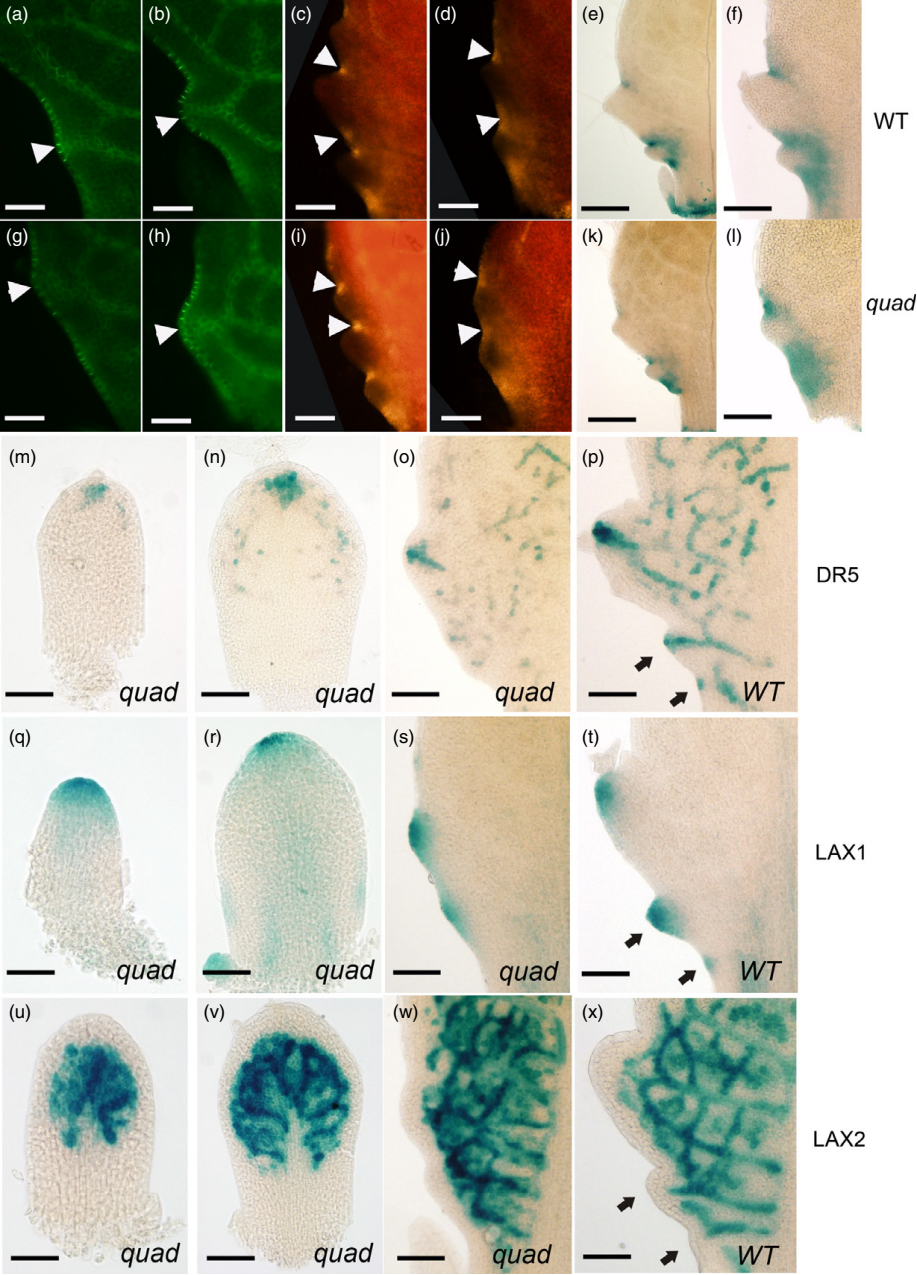
*PIN*,*CUC2*,*DR5* and *LAX1* gene expression patterns define serrations in WT and *quad* mutant leaves. (a–l) Expression patterns of Pro_PIN__1_:GFP (a,b,g,h), Pro_CUC__2_:RFP (c,d,i,j) and Pro_CUC__2_:GUS (e,f,k,l) in serrations forming on the margin of WT (a–f) and quadruple *aux1*/*lax* mutant (*quad*) leaves (g–l) at various stages of serration formation. In (a,b,g,h), arrowheads indicate the tip of early‐stage serrations. In (c,d,i,j), arrowheads indicate the sinus between serration outgrowths. The Pro_PIN__1_:GFP signal is asymmetrically localized in cells, suggesting a flow of auxin towards the tip of serrations, whereas CUC2 expression is localized within the sinuses of serrations in both genetic backgrounds. (m–p) Pro_DR__5_:GUS expression (blue) in the *quad* mutant background at the early‐stage (m) and mid–stage (n) of leaf 5 development was observed at the tip of the primordium, and, at a later stage of development (o), at the tip of serrations. Pro_DR__5_:GUS expression in WT is shown for comparison (p). (q–t) Analysis of Pro_LAX__1_:GUS leaves shows a comparable expression pattern at the leaf tip during the early‐stage (q) and mid‐stage (r) of development, with signal (blue) also apparent within the serrations that form later in development of the *quad* leaves (s) compared to WT (t). (u–x) The images in (u), (v) and (w) show stages of development equivalent to those in (m), (n) and (o), respectively, for Pro_LAX__2_:GUS in a *quad* background. Signal is constrained to the more distal region of the leaf during early development, and is excluded from the outer cell layers at all stages of development, as also seen in WT serrations (x). Arrows in (p), (t) and (x) indicate small serrations. Exemplar images are shown from the analysis of at least six independent plants for each reporter gene construct in each genetic background. Scale bars = 50 μm (a–m,r,v), 100 μm (n,r,v) and 80 μm (o,p,s,t,w,x).

Previous work established that PIN1 and CUC2 play a major role in serration formation, with PIN protein localization indicating auxin flux towards presumptive serration tips and CUC2 accumulation indicating intervening sinuses (Bilsborough *et al*., 2011). An examination of PIN1 and CUC2 expression using Pro_PIN1_:PIN1‐GFP, Pro_CUC2_:CUC2‐RFP and Pro_CUC2_:GUS transgenes revealed no differences in the expression patterns of these proteins in the *quad* mutant compared to WT (Figure 3). Thus, although the final extent of serration growth was less in the *quad* mutant, the patterning process was comparable in both the WT and *quad* mutant background. Thus, whenever a serration formed along the margin, the position of the serration outgrowth was defined by PIN1 orientation on the flank of the presumptive outgrowth pointing towards the lobe tip, both in the WT (Figure 3a,b) and the *quad* mutant (Figure 3g,h). Similarly, CUC2 expression was elevated in the sinuses between serrations in both WT (Figure 3c–f) and *quad* mutant leaves (Figure 3i–l), as visualized using RFP (Figure 3c,d,i,j) and GUS (Figure 3e,f,k,l) reporter gene lines. With respect to Pro_DR5_:GUS expression, in the early stages of primordium development of *quad* plants, the signal was apparent at the tip of the leaf (Figure 3m), and, as development proceeded, signals appeared later along the flanks of the primordia, but only as serrations were formed (Figure 3o), and the signal was broader and weaker compared with the WT expression pattern (Figure 1d). *LAX1* promoter activity was still high at the tips of early and mid‐stage primordia in the *quad* background (Figure 3q,r) and in regions of serration tip formation (Figure 3s), comparable to the pattern observed in WT (Figure 3t), although the altered growth rate of tissues in the various genetic backgrounds resulted in different absolute distances of peak formation. Investigation of *LAX2* expression in the *quad* background showed that the pattern of gene expression was similar to that observed in WT leaves at equivalent developmental stages, with *LAX2* expression being initially constrained towards the distal leaf tip but excluded from the outer cell layers of the margin at all stages of development (Figure 3u–x).

**Figure 4:**
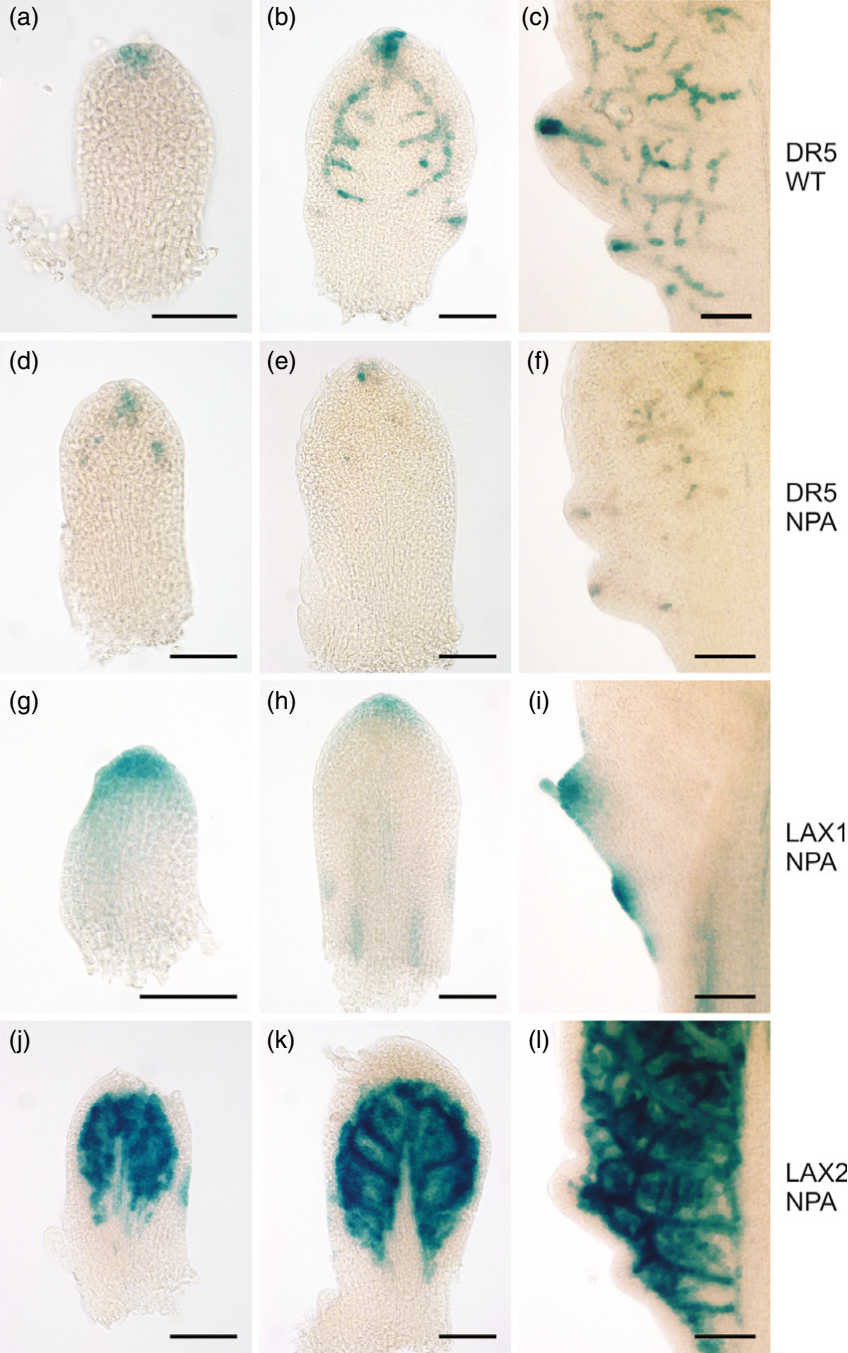
*DR5*,*LAX1* and *LAX2* expression patterns in leaves after N‐1‐Naphthyphthalamic acid (NPA) treatment. Analysis of Pro_DR__5_:GUS (a–f), Pro_LAX__1_:GUS (g–i) and Pro_LAX__2_:GUS (j–l) expression in early‐stage (a,d,g,j), mid‐stage (b,e,h,k) and late‐stage (c,f,i,l) primordia. (a–c) Analysis for control plants; (d–l) analysis for plants treated with 1 μm NPA. Exemplar images are shown from the analysis of at least six independent plants for each reporter gene construct line after treatment with NPA. Scale bars = 50 μm.

In addition to the use of mutants, auxin transport may be manipulated by exogenous supply of inhibitors, and previous reports indicated that treatment of Arabidopsis plants with NPA (N‐1‐Naphthyphthalamic acid) leads to a smoother leaf margin (Mattsson *et al*., 2003). Control plants showed a normal pattern of serration during development, with P_DR5_:GUS expression at the primordium tip (Figure 4a), at the tips of serrations and in portions of an internal network (Figure 4b,c). When NPA was supplied to plants at 1 μm, serration was inhibited in a manner similar to that described for the *aux1*/*lax1*/*lax2* and *quad* mutants (i.e. serration still occurred and was still restricted to the base of the leaf, but it occurred later than in the untreated control leaves) (Figure 4d–l). Analysis of Pro_DR5_::GUS expression in NPA‐treated leaves indicated the presence of an appropriate pattern of auxin maxima at the presumptive tips of serrations but with decreased signal intensity (Figure 4d–f). After NPA treatment, *LAX1* gene expression occurred at the tips of serrations, although, as described above, these serrations were less pronounced than in non‐treated leaves (Figure 4g–i). The pattern of *LAX2* gene expression in NPA‐treated leaves was similar to that observed in control leaves, being restricted towards the distal region of the leaf and excluded from the outer cell layers (Figure 4j–l).

### Modelling the role of auxin importers in the leaf margin

**Figure 5:**
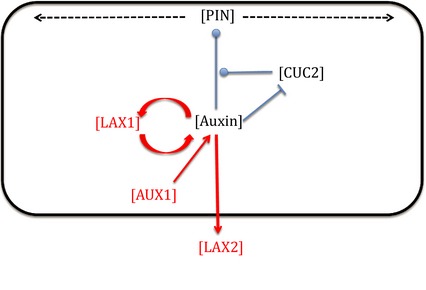
AUX/PAT/CUC2 model for margin patterning. The concentration of auxin regulates the localization of PIN auxin exporters within a cell, directing them to either the left or right border using an ‘up the gradient’ rule with respect to neighbouring cells. The process by which auxin modulates PIN localization is modulated by the CUC2 transcription factor, the level of which is inversely correlated with auxin level. The level of LAX1 is positively regulated by the level of auxin, which is itself positively regulated by the LAX1 level in a positive feedback loop. Auxin is drained from the system by LAX2, and this drain is counteracted by auxin influx via AUX1. Details of the model are provided in Model S1. Items in black represent the model parameters included in the original model (Bilsborough *et al*., 2011), and the items in red indicate the new parameters included in the AUX/PAT/CUC2 model.

To further explore how the patterns of *AUX1*/*LAX* gene expression relate to the observed phenotypes, we used a modelling approach. Our initial model (model variant A) was adapted from that described by Bilsborough *et al*. (2011) but modified to include rules based on the *AUX1*/*LAX* gene expression patterns reported above and in the literature, and simplified with respect to assumptions regarding PIN localization. As shown in Figure 5, we first introduced a positive feedback loop of auxin import linked to the local auxin concentration, representing both the observed correlation of *LAX1* expression with regions of localized DR5 expression along the leaf margin and other data suggesting a link between auxin import and auxin levels (Heisler and Jonsson, 2006). Second, we imposed a drain of auxin from margin cells in a region symmetrically positioned around the leaf distal tip to simulate the outcome of the observed initial localization of *LAX2* expression in a distally located sub‐epidermal region of the leaf during the early stages of leaf growth. Third, we set an initially uniform auxin import rate set along the entire perimeter to reflect the observed *AUX1* expression pattern at the early stage of primordium formation. Details of this model are described in Model S1.

**Figure 6:**
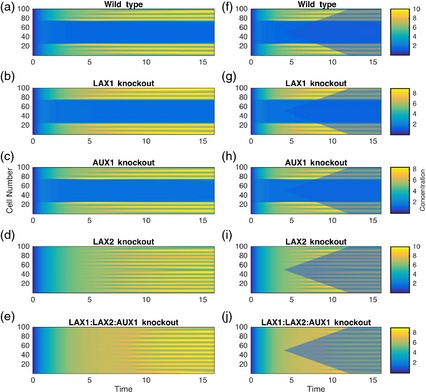
Analysis of the AUX/PAT/CUC2 model. Space/time displays of auxin levels as output of an AUX/PAT/CUC2 model in which the leaf margin is depicted as a row of cells (*y* axis) with the extremities of the margin at cell 1 and cell 100 and the distal tip of the leaf margin at cell 50. Auxin level is indicated by a spectrum of colour from low (blue) to high (yellow). (a) The basic model in which auxin peaks are restricted to the margin periphery and excluded from the central region of the margin. Auxin peaks initiate at time point 2.5, with the last peak initiating at time point 5. (b,c) A *lax1* mutant model (b) and an *aux1* mutant model (c) in which the overall pattern of auxin peaks (both in space and time) remains similar to that shown in (a). (d) Loss of *LAX2* leads to the emergence of auxin peaks along the entire margin and a delay in formation of the first auxin peak. (e) In the *aux1*/*lax1*/*lax2* mutant model, auxin peaks occur along the entire margin, but the emergence of the auxin pattern is greatly delayed. (f) A modified version of the model shown in (a) in which an auxin sensitivity window is imposed, indicated by the translucent blue coloration. Loss of auxin sensitivity occurs first at the most distal margin cell (cell 50) at time point 2. Over subsequent iterations, an adjacent margin cell loses auxin sensitivity, leading to linear loss of auxin sensitivity over time until all margin cells become insensitive to auxin by time point 10. (g–j) Introduction of the auxin sensitivity window to the model for mutant *lax1* (g), *aux1* (h), *lax2* (i) and *aux1*/*lax1*/*lax2* (j). In some cases (g,h,i), auxin peaks emerge for a distinct period before the loss of auxin sensitivity, whereas in others (j), auxin peaks occur for only a brief period before auxin sensitivity is lost. If the integral of auxin level (yellow peak value) is proportional to local growth, then the models lead to differential serration growth.

The outputs of the model are displayed as space/time plots (Figure 6). In these plots, the leaf margin is represented as a line of cells with the margin extremities at the top and bottom of the y axis and the distal tip, equivalent to the margin mid‐point, located at the mid‐point of the *y* axis (cell 50). Auxin concentration is depicted as a colour spectrum, with maxima indicated by regions of bright yellow and troughs as dark blue. As in the original model, we make the assumption that a local gradient in auxin level is somehow read out by the cells in that region as a gradient in growth response, leading to initiation of morphogenesis (i.e. serration). The extent of growth (i.e. serration size) is not explicitly modelled, but is assumed to be proportional to the integrated level of auxin.

This model generates a symmetrical pattern along the perimeter with alternating peaks and troughs of auxin level (Figure 6a). The expression of *LAX2* around the leaf tip drains auxin away from the margin in this region, ensuring that no auxin maxima are formed. This is predicted to lead to a lack of serrations in this region, as observed in WT Arabidopsis leaves (Figure 2d,g). Interestingly, the *LAX2*‐defined drain generates an internal boundary within the margin, and, as a consequence, peaks of auxin form in a temporal sequence, with peaks towards the leaf tip occurring before peaks at the leaf base. Thus the first auxin peak forms at approximately time point 2.5, and the last auxin maximum occurs at approximately time point 5. This reproduces the situation observed in real leaves, in which serration occurs with a pronounced distal‐to‐proximal temporal pattern.

The model presented in Figure 6(a) captured some elements of margin patterning that were not apparent in the original model. The precise timing of peak occurrence and the relative intensity of the peaks depended on the values given to the various parameters, but the basic output (lack of peaks in the distal region and the temporal sequence of peak formation from distal to proximal region) was consistent. The model did not automatically generate a peak of auxin at the distal tip of the leaf, but analysis of auxin‐response constructs in transgenic plants consistently indicates that this is present (Mattsson *et al*., 2003). A significant body of evidence suggests that this initial peak of auxin signalling is intrinsically linked to the process of leaf initiation, i.e. it is already set within the leaf at formation (Reinhardt *et al*., 2003). Although we imposed this central auxin peak within early versions of our model, we found that its presence had little effect on subsequent model behaviour, so, for simplicity, it is not included in the variants shown.

To investigate how well the model captures the phenotypes observed in mutants, we explored the outcome of removal of components of the model. As shown in Figure 6(b,c), removal of either the *LAX1* or *AUX1* components had relatively little effect on model output for each parameter set. Patterning was slightly delayed in the proximal regions of the margin compared with the WT model (Figure 6a), but the distribution and timing of auxin peak formation was essentially unchanged. This relatively limited outcome of removal of the *LAX1* and *AUX1* components was consistent with the observed *lax1* or *aux1* single mutations, in which no obvious change in serration was observed. However, when *LAX2* was removed from the model (as shown in Figure 6d for the *lax2* mutant and Figure 6e for the *aux1*/*lax1*/*lax2* mutant), a fundamental change in patterning occurred. First, and most obviously, auxin peaks were predicted to occur throughout the margin, including the distal region. This would lead to serration being initiated in the distal leaf margin, but this phenotype was never observed in the *lax2* or *aux1*/*lax1*/*lax2* mutant leaves. Another consequence of the loss of *LAX2*, which was most apparent in the *aux1*/*lax1*/*lax2* mutant model and to a lesser extent in the *lax2* model, was that the time taken for auxin patterning to be established was greatly delayed (Figure 6d,e). Again, the absolute time taken for peaks to emerge depended on the parameters used, but the results were consistent for each set of parameters explored. Thus, in the *lax2* mutant model shown in Figure 6(d), auxin peaks occurred uniformly at approximately time point 5, and, in the *aux1*/*lax1*/*lax2* mutant model (Figure 6e), auxin peaks did not arise until approximately time point 7, much later than the slowest‐forming auxin peaks in the WT and *lax2* models (Figure 6a).

### Reconciling model and reality: exploring the roles of auxin sensitivity and leaf growth rate

The model described above has a major discrepancy with respect to one aspect of the biological phenotype observed. In reality, mutants containing *lax2* do not form serrations in the distal region of the leaf. To investigate the possible reason for this discrepancy, we considered the potential role of altered auxin sensitivity during development. It is noteworthy that differentiation of margin cells is one of the earliest observable events in leaf development, and, moreover, that it occurs in a temporal wave from the distal tip towards the proximal regions of the margin (Reinhardt *et al*., 2007). Whether this overt early margin differentiation is linked to altered auxin sensitivity is unknown, but links between auxin signalling and the cell cycle are well‐established (Menges *et al*., 2005; De Veylder *et al*., 2007; Jurado *et al*., 2010). To explore this idea, we imposed a temporal wave of auxin insensitivity on the models described in Figure 6(a–e) (model variant B). The outputs shown in Figure 6(f–j) provide examples based on the assumption of a simple linear loss of auxin sensitivity with time, starting at the most distal (tip) margin cell at time point 2 and progressing towards the proximal region at a set rate such that the entire margin becomes insensitive with respect to the auxin patterning system by time point 10. This pattern of sensitivity loss is clearly arbitrary, but serves as an example of the type of outputs that may result from such sensitivity windows.

For the WT, *lax1* and *aux1* models, the outputs are very similar after imposition of the auxin sensitivity window (Figure 6f–h). If the assumption is made that growth of the resulting serrations depends on the integrated level of auxin over time within any one peak before sensitivity is lost, then the relative size and position of peaks would be similar, as observed in reality. When a sensitivity window is included in the model for the *lax2* mutant (Figure 6i), peaks of auxin are also still formed in the proximal regions of the margin ahead of the wave of auxin insensitivity in positions similar to WT, such that absolute peak number and position are conserved. The integrated level of auxin within a peak is less than in the model outputs shown in Figure 6(f–h), and therefore the serration growth is predicted to be less in this mutant than in WT or the other single mutants, although the degree of difference depends on the parameter values set. In the *aux1*/*lax1*/*lax2* mutant model (Figure 6j), the loss of all auxin importer activity results in most of the margin losing auxin sensitivity before auxin peaks are established, leading to integrated auxin peak levels being very small (although they still occur in the appropriate positions). Again, if the assumption is made that serration growth depends on the integral of auxin level at a position on the margin, it may be predicted that serration in this mutant still occurs but that the serrations would be much smaller, as indeed observed in the triple and quadruple mutants.

Addition of NPA (auxin transport inhibition) to the model without the sensitivity window led to a similar output to that observed for the *quad* mutant (Figure S2A versus Figure 6e) with the effect depending on the degree to which the transport system was inhibited (equivalent to the concentration of NPA supplied to the tissue) (Figure S2B,C). Incorporation of the auxin sensitivity window meant that there was still potential for auxin peaks to form for a brief time towards the margin periphery (thus some degree of serration) (Figure S2D), or, if auxin transport was severely inhibited (equivalent to very high levels of NPA), total loss of auxin patterning along the leaf margin (Figure S2E,F). Again, if serration growth is related to the integral of the auxin peak level before the loss of auxin sensitivity, the final serration size will be decreased as a result of NPA treatment.

### Testing the model reveals a role for margin‐localized auxin signal transduction in blade/petiole growth

**Figure 7:**
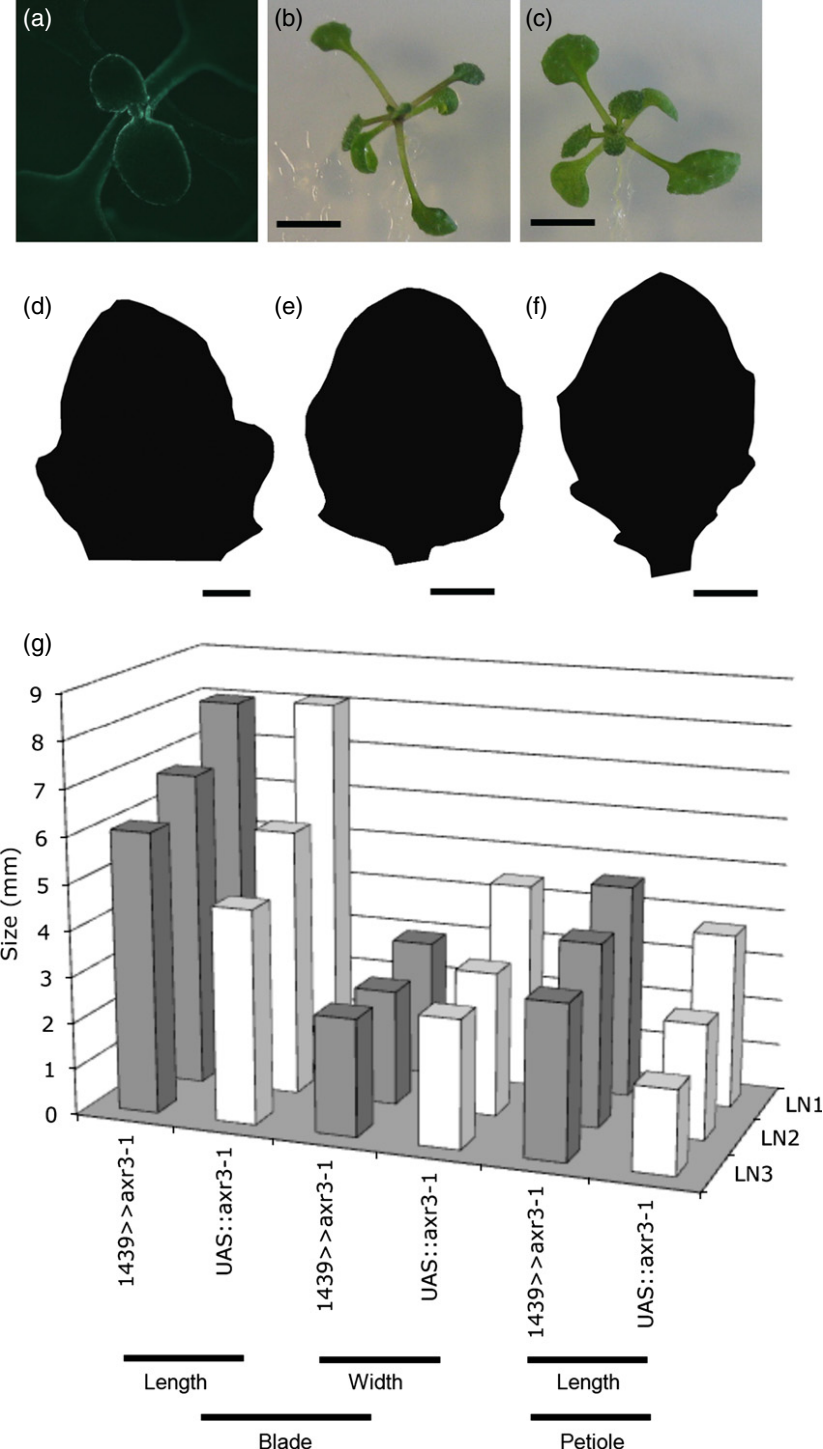
Testing the role of auxin signalling in the leaf margin. (a) E1439 drives GFP and target gene expression to the leaf margin and petiole. (b,c) E1349 > axr3–1 plants (b) show a phenotype of relatively long petioles and smaller blades compared with control E1439 plants (c). Scale bars = 5 mm. (d–f) Exemplar images of young (d), mid‐stage (e) and late‐stage (f) E1439 > axr3–1 leaves show the presence of serrations (observed in six independent plants). Scale bars = 100 μm (d), 200 μm (e) and 500 μm (f). (g) Quantification of petiole and blade size of leaves from E1439 > axr3–1 plants and control UAS::axr3–1 plants, revealing differences in size and form. The results are shown for experiments performed with three independent lines of E1439 > axr3–1 (LN1, LN2, LN3), and the progeny of three crosses of UAS::axr3–1 plants with Col–0 WT plants. Mean values for the parameters measured (blade length and width, petiole length) are shown, with measurements from leaves of 12 individual plants in each case. A Student's *t* test comparing petiole length in E1439 > axr3–1 versus UAS::axr3–1 leaves indicated a significant difference (*P *< 0.01) (*n* = 3).

The model developed above introduces a potential role of changing auxin sensitivity in serration formation and growth. To test this hypothesis, we used a previously characterized enhancer trap line (E1439) that drives expression of target genes in a dynamic fashion to the leaf margin (Reinhardt *et al*., 2007). Expression first occurs in the distal margin, and then extends around the complete margin and petiole as the leaf develops (Figure 7a). We used the E1439 line to drive expression of a gain‐of‐function mutation in *axr3–1* (*IAA17*). This Aux/IAA protein has been shown to alter various plant responses to auxin, although the precise nature of the downstream process (in terms of increased or decreased auxin sensitivity) is complex and is likely to be context‐dependent (Leyser *et al*., 1996; Perez‐Perez *et al*., 2010). The E1439 > axr3–1 plants displayed an unexpected phenotype at the whole‐organ level. The ratio of the petiole to leaf blade was increased, leading to rosettes with greatly elongated petioles and relatively narrow blades compared with control UAS::axr3–1 plants (Figure 7b,c). Quantitative analysis of various leaf size parameters (Figure 7g) confirmed this visual impression, with E1439 > axr3–1 leaves having a significant (*P *<* *0.01) increase in petiole length. There was also a change in blade shape, with E1439>axr3–1 blades being relatively more elongated than the controls. Nevertheless, serrations did form on the E1439 > axr3–1 leaves, with the pattern of serration being comparable to that of WT leaves (Figure 7d–f).

## Discussion

The role of auxin transport in patterning in plants has been investigated in detail (Vanneste and Friml, 2009), and a general conclusion is that *AUX1*/*LAX* importer proteins play an important role in stabilizing patterns initiated via the PAT system, such as leaf initiation (Kramer, 2004; Heisler and Jonsson, 2006; Bainbridge *et al*., 2008; Peret *et al*., 2013). Our data indicate that the *AUX1*/*LAX* system also plays a role in determining the degree of serration around the leaf perimeter. Thus, loss of activity of all three *AUX1*/*LAX* importers normally expressed in the developing leaf leads to a phenotype in which serration size is decreased. Interestingly, this phenotype was not apparent in single or double mutant combinations, despite the individal genes showing distinct expression patterns. This suggested a non‐intuitive combinatorial spatial influence of auxin import on serration rather than simple genetic redundancy. Use of transcriptional and translational promoter fusions suggested that the basic PIN1/CUC2 patterning process was still occurring in the *aux1*/*lax1*/*lax2* and *quad* mutants, but the intensity of auxin signalling (as estimated using the DR5 reporter system) was decreased. At the same time, the extent of differential growth (which underpins serration) was also decreased. These results led us to explore the potential mechanism by which the complex and dynamic pattern of *AUX1*/*LAX* importer proteins influences differential growth via altered accumulation of auxin.

To do this, we used a modelling approach. Building on an established model, we incorporated the new expression data by linking LAX1 activity to the accumulation of auxin (using DR5 expression as a proxy), interpreting the *LAX2* expression pattern as a drain of auxin out of the system in a spatially defined manner set by the observed expression pattern, and setting import into the system as uniform based on the observed pattern of *AUX1* expression. This led to a model output that recapitulated the observed pattern of serration in real leaves, i.e. an absence of serrations in the distal portion of the leaf and a temporal sequence of serration formation from the *LAX2* boundary towards the proximal region of the leaf. However, although the model accounted for some phenotypes (e.g. single and combinatorial mutants of *AUX1* and *LAX1*), it failed to provide an accurate representation of mutants involving *LAX2*.

One possible solution reason for this is that there is a temporal control of sensitivity such that the system either loses the ability to respond to auxin peaks over time or loses the ability to generate auxin peaks over time. Alternatively, a shift in relative growth rate along the proximal/distal axis over time may account for the restriction of serrations to the base of the leaf. These two possibilities are considered below.

To assess the first option, we incorporated a sensitivity window into our model to see whether this provided a conceptual solution to the problem. Imposition of a simple linear temporal decline in auxin responsiveness suggested why a limited outcome on serration is observed in single and double mutants, whereas in the triple mutant containing the *lax2* allele, the imposed sensitivity window had a major outcome on serration due a decrease in the time during which auxin accumulation occurs before sensitivity to auxin with respect to growth is lost. The existence of such a sensitivity window is of course speculative, but there are some observations to support it. For example, margin cells undergo a very early and dramatic increase in cell size during leaf development, this differentiation occurs in a temporal wave from the leaf tip around the leaf perimeter towards the leaf base, and ablation of these cells abrogates leaf development (Zgurski *et al*., 2005; Reinhardt *et al*., 2007). Whether this early overt differentiation is linked to a change in auxin sensitivity is unknown, but there is extensive evidence linking auxin to the cell cycle and the decision to continue proliferation or exit towards expansion (Braun *et al*., 2008; Jurado *et al*., 2010). In addition, it has been observed that PIN1 expression is gradually lost from the margin (Wenzel *et al*., 2007), and this would also probably lead to loss of the ability of these cells to form auxin‐response maxima.

Our attempts to test the sensitivity model via enhancer trap‐directed expression of *axr3–1* (*IAA17*) were inconclusive. Expression of *axr3–1* in the margin led to a change in global form of the leaf (increased petiole length and decrease in blade size). Within these blades, some serration occurred, arguing against the sensitivity model. However, this interpretation must be treated with caution given that that the *axr3–1* gene is known to be a relatively crude tool for manipulation of auxin responsiveness, and that it is necessary to be careful in distinguishing between serration initiation and subsequent growth. For example, the *iaa8*/*9* double mutant has disrupted auxin signalling (presumably throughout the leaf), and this leads to a phenotype in which, although serrations are initiated, subsequent changes in growth distibution lead to a relatively smooth margin (Koenig *et al*., 2009). Auxin signalling appears to be involved in both serration initiation and outgrowth, but our understanding of the signalling mechanism linking auxin to these outputs remains limited and thus remains an area of intense research (Barbez *et al*., 2012; Peer, 2013; Paque *et al*., 2014).

The readout of auxin signalling into growth may vary during development, and it is entirely plausible that the oberved restriction of serration to the leaf base reflects a gradient of growth rate along the leaf proximal/distal axis rather than an inferred change of auxin sensitivity limited to the margin. Although the few measured areal growth rates available do not indicate massive growth differentials along the proximal/distal axis at the time the first serrations are forming, significant differential growth rates do subsequently appear (Kuchen *et al*., 2012). These growth rate transitions are accompanied by a wave of cell cycle exit along the proximal/distal axis (Nath *et al*., 2003), leading to cell division becoming gradually restricted towards the leaf base where serration initiation is occuring. Plant cells are distinguished by transition from cell division‐associated to non‐cell division‐associated growth as they exit the cell cycle (Fleming, 2006). Non‐cell division‐associated growth is often linked to a faster relative cellular growth rate driven by vacuolar expansion, but the final extent of growth (size of an organ or serration) is to a large extent dependent on the earlier investment of cell divisions to generate a body of cells that each undergo an expansion phase. In the context of serration, initial formation of a serration requires some ability for cell division in that region, but the subsequent relative size and shape of the serration depend upon the relative growth rate (and thus the cell‐division exit trajectory) of the cells both within and surrounding the serration initiation site. There are very few experimental growth data at this resolution coupled with estimates of cell division rate at the leaf margin (Kawamura *et al*., 2010). Recent work on *Eschscholzia* indicated that, in this system, there are gradients of relative growth rate along the proximal/distal axis of the leaf in the region where leaflets are being formed in the compound leaf, consistent with the proposal that lateral outgrowths occur in an acropetal direction due to the realtively high growth rate of the distal part of the leaf (Ikeuchi *et al*., 2014). In Arabidopsis, serrations occur in a basipetal direction, with the first outgrowths occurring approximately halfway along the primordium. The lack of serrations in the distal tip of the leaf may reflect the fact that these cells have exited from the cell cycle and thus are unable to initiate the cell divisions required for the future growth underpinning morphogenesis. The interaction of cell division, exit from the cell cycle and growth in plants is clearly complex, and our work highlights the need for further measurement of these parameters at the appropriate resolution to resolve the various contributions made to shape change. The nature of these interactions varies with time along the distal/proximal axis of the leaf, and is closely linked to the program of cellular maturation, but what controls the rate of maturation remains a key open question for future research (Andriankaja *et al*., 2012; Hepworth and Lenhard, 2014).

## Experimental procedures

### Plant material and growth

All *Arabidopsis thaliana* lines were in the Columbia background, and plants were grown as described by Kuwabara *et al*. (2011). Briefly, seeds were kept at 4°C for 1 week before sowing on 0.8% w/v agar medium containing half‐strength MS salt mix (Sigma, www.sigmaaldrich.com) and 1% w/v sucrose. Seedlings for which leaf number 5 was approximately the same size (measured under a stereomicroscope) were selected after 10 days and used for experimentation. Growth conditions were 100 μmol m^−2^ sec^−1^ light, a 16/8 h photoperiod, and temperature 20/18°C (light/dark). The mutant lines *aux1*,* lax1*,* lax2*,* lax3* and their combinations have been described previously, as have the Pro_AUX1_:GUS, Pro_LAX1_:GUS, Pro_LAX2_:GUS and Pro_LAX3_:GUS lines (Bainbridge *et al*., 2008; Peret *et al*., 2012). Pro_DR5_:GUS has been described previously (Mattsson *et al*., 2003). The Pro_PIN1_:GFP line (a gift from J. Friml, Institute of Science and Technology Austria, Vienna, Austria) was crossed with both the triple *aux1*/*lax1*/*lax2* and quadruple *aux1*/*lax1*/*lax2*/*lax3* lines, with homozygous Pro_PIN1_:GFP lines being confirmed by PCR. The Pro_CUC2_:GUS line was a gift from Patrick Laufs (INRA Versailles, France), and has been described previously (Nikovics *et al*., 2006; Bainbridge *et al*., 2008). The Pro_CUC2_:RFP line was produced and kindly provided by Hasson and Laufs (unpublished). They were crossed into the *triple aux1*/*lax1*/*lax2* and quadruple *aux1*/*lax1*/*lax2*/*lax3* lines, and homozygous lines were identified by PCR. PCR for specific *AUX1*/*LAX* genes was performed using the following primers: *LAX1*; 5′‐ATATGGTTGCAGGTGGCACA‐3′ and 5′‐GTAACCGGCAAAAGCTGCA‐3′; *LAX2* 5′‐ ATGGAGAACGGTGAGAAAGCAGC‐3′ and 5′‐CGCAGAAGGCAGCGTTAGCG‐3′; *LAX3* 5′‐ TACTTCACCGGAGCCACCA‐3′ and 5′‐TGATTGGTCCGAAAAAGG‐3′. The E1439 > axr3–1 lines were created by first cloning the *axr3–1* cDNA under the control of a UAS promoter to generate a UAS::axr3–1 construct that was transformed into Col–0 WT plants, as previously described (Reinhardt *et al*., 2007). Homozygous lines were selected by selection on antibiotic‐containing medium, and T_3_ progeny were crossed with either the homozygous E1439 enhancer trap or WT plants as a control. Progeny were selected based both on antibiotic selection and fluorescence microscopy, revealing the margin‐specific GFP expression pattern of the enhancer trap. At least 12 plants from each of three independent lines were used in subsequent growth analysis.

### Analysis of gene expression and mutant phenotypes

For GUS histochemical analysis, plants were pre‐treated with 90% ice‐cold acetone, and further assay was performed according to established protocols (Jefferson *et al*., 1986). The substrate solution contained 5 mM each of potassium ferricyanide and ferrocyanide. After clearing in chloral hydate (Kuwabara *et al*., 2011), images were taken using a DP71 camera (Olympus, http://www.olympus.co.uk/) mounted on a BX51 light microscope (Olympus) or SZ12 stereomicroscope (Leica, http://www.leica-microsystems.com/). GFP and RFP fluorescence observation were performed using a BX51 microscope with 470‐490 nm excitation and a 515‐550 nm barrier filter (narrow GFP band‐pass), or 330‐385 excitation and a 420 nm long‐pass filter. For leaf shape changes, observations were made on at least ten plants per line. Individual leaves (leaf 5) from staged plants were removed and imaged as described by Kuwabara *et al*. (2011).

### Leaf shape analysis

Images of dissected leaves (leaf 5) were imported into the LeafProcessor software program (Backhaus *et al*., 2010), which provides a semi‐automatic and landmark‐free method for analysis of a range of leaf‐shape parameters. The compactness parameter provides a scale‐free measure of the ratio of leaf perimeter length to enclosed area (*P* ^2^:*A*). For bending energy, at each sample point along the contour, a curvature value is calculated that is then squared and integrated along the contour, providing a scale‐free global measure of the curvature of the leaf perimeter. At least three independent leaf samples for each developmental stage and each genotype were analysed using LeafProcessor. In addition to use of the statistical package within the software, data were also exported to Prism 6 (http://www.graphpad.com) for statistical analysis and chart drawing.

### Modelling

We developed a computational model to test the effects that the AUX1/LAX family of auxin influx importers have on leaf margin development. This model is adapted from that described by Bilsborough *et al*. (2011), but no assumptions on pre‐existing PIN polarity are incorporated. The model is described in detail in Model S1. Briefly, the leaf margin is simulated as a one‐dimensional file of 100 cells. Each cell has a concentration of auxin, LAX1, LAX2, CUC2 and PIN1. PIN1 is preferentially allocated to the cell walls, according to an established formalism (Smith *et al*., 2006). We assume that auxin peaks promote LAX1 expression, which in turn amplifies these peaks by enabling the influx of auxin. At early developmental stages, LAX2 is only expressed in the distal half of the leaf, and we assume that this acts to draw auxin away from the leaf margin, preventing auxin peaks from forming. AUX1 is expressed equally around the leaf perimeter. Simulations start with equal levels of auxin and proteins in all cells, with the addition of a small amount of noise to break symmetry. The dynamics of auxin and the four types of protein in each cell are modelled by a series of ordinary differential equations, details of which are provided in Model S1, which also gives information on the parameter values selected. Simulations were run until a steady state was achieved. Models were implemented in MATLAB version 7.14 (MathWorks, http://uk.mathworks.com/).

## Supporting information


**Figure S1.** Mutations in pairs of auxin importers do not lead to a delay in serration.

**Figure S2.** AUX/PAT/CUC2 model of pattern formation after treatment with NPA.

**Model S1.** Description of the mathematical model.
