# Supplementary figures and images for: Auxin influx importers modulate serration along the leaf margin
Source: Plant J. 2015 Jul 27;83(4):705–18. doi: 10.1111/tpj.12921 (PMC4949643; doi:10.1111/tpj.12921)

*aux1/lax1*

*aux1/lax2*

*aux1/lax3*

(a)

(b)

(c)

Early

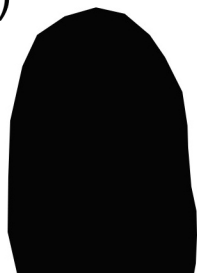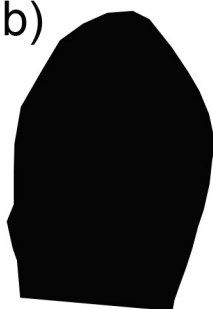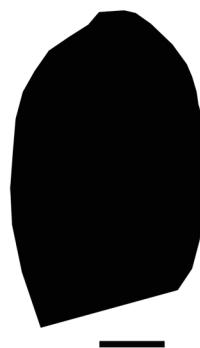

(d)

(e)

(f)

Mid

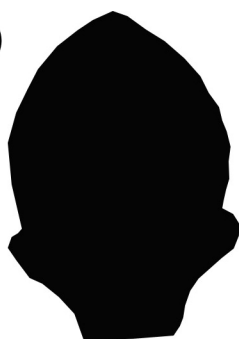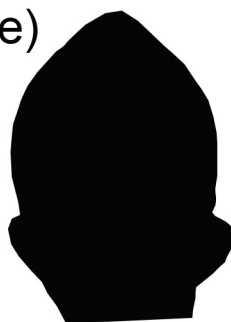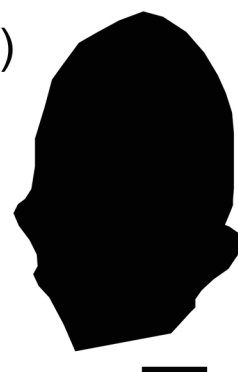

(g)

(h)

(i)

Late

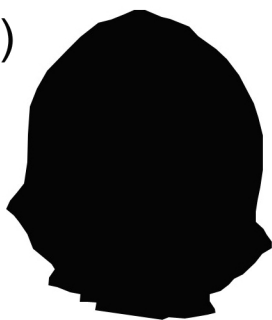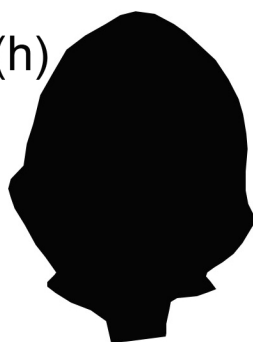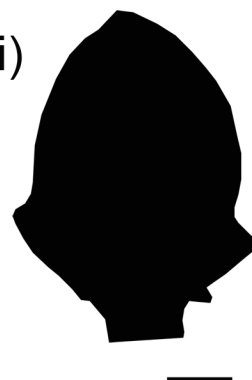

Supplement: Supplementary file 1 — Figure S1. Mutations in pairs of auxin importers do not lead to a delay in serration. [file TPJ-83-705-s001.pdf]

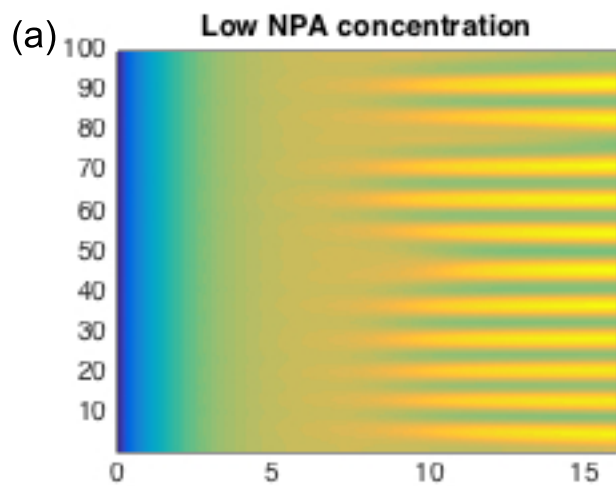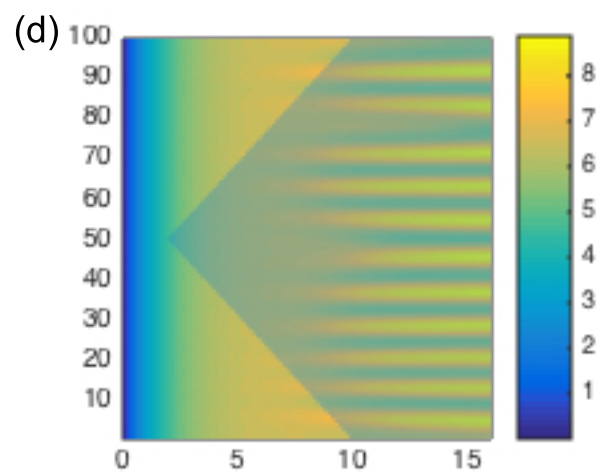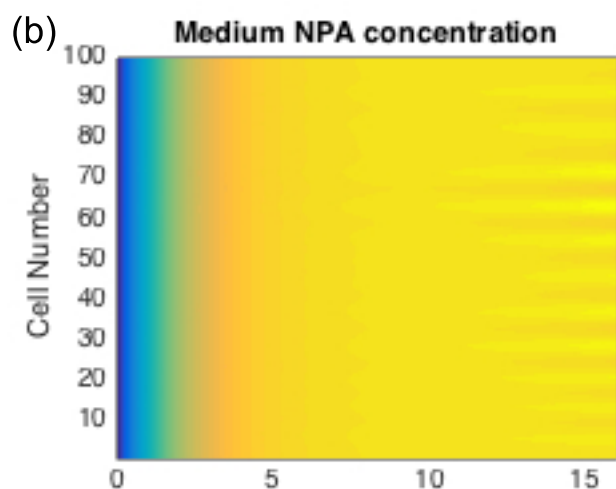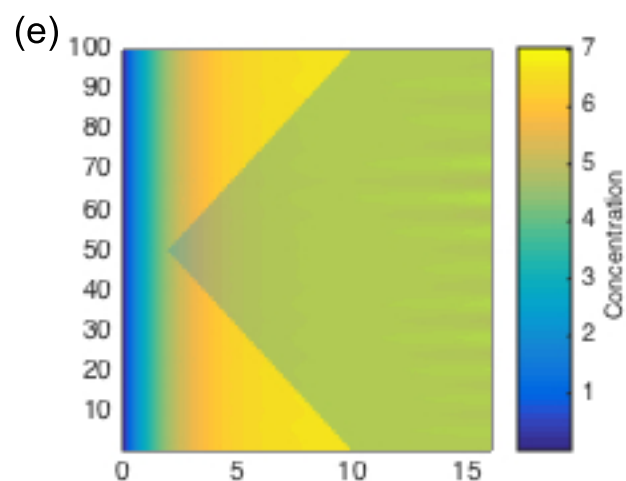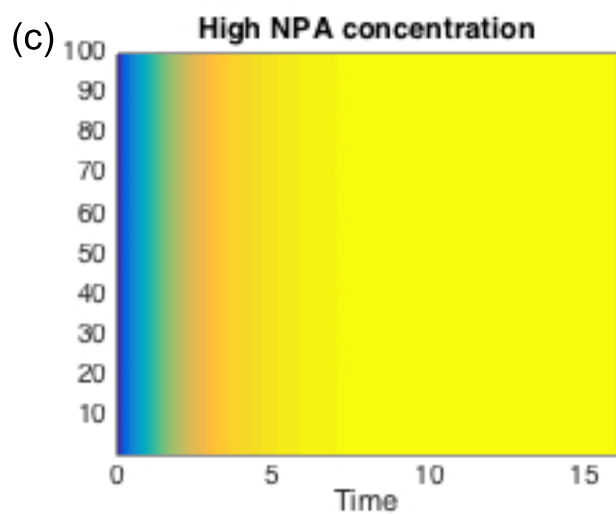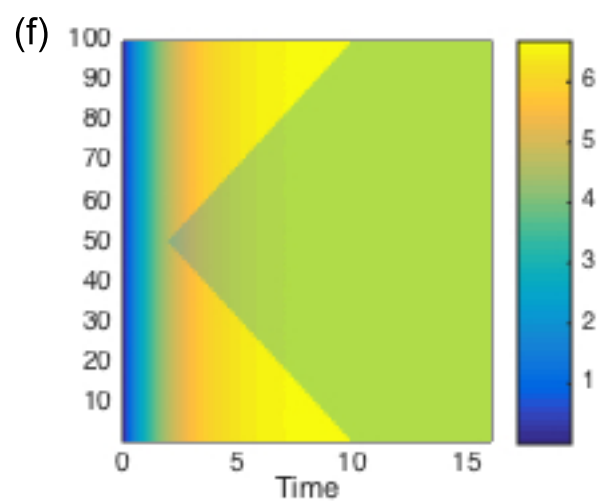

Supplement: Supplementary file 2 — Figure S2. AUX/PAT/CUC2 model of pattern formation after treatment with NPA. [file TPJ-83-705-s002.pdf]
