# Supplementary material for: Auxin influx importers modulate serration along the leaf margin
Source: Plant J. 2015 Jul 27;83(4):705–18. doi: 10.1111/tpj.12921 (PMC4949643; doi:10.1111/tpj.12921)
Supplement: Supplementary file 3 — Model S1. Description of the mathematical model. [file TPJ-83-705-s003.pdf]

Description of the Mathematical Model

The system described by this model is a one dimensional row of N cells which represents the cells contained within the leaf boundary. Each cell is labelled with an index number, i, where $1 \leq i \leq N$. The cells 1 and N represent the cells adjacent to the petiole (Fig. 1). In this model the size of the cells and the cell walls is fixed to unity and the geometry is assumed to be static. When modelling transport in the system the apoplectic space is ignored and transported auxin is assumed to be transported directly between the cytosol of neighbouring cells.

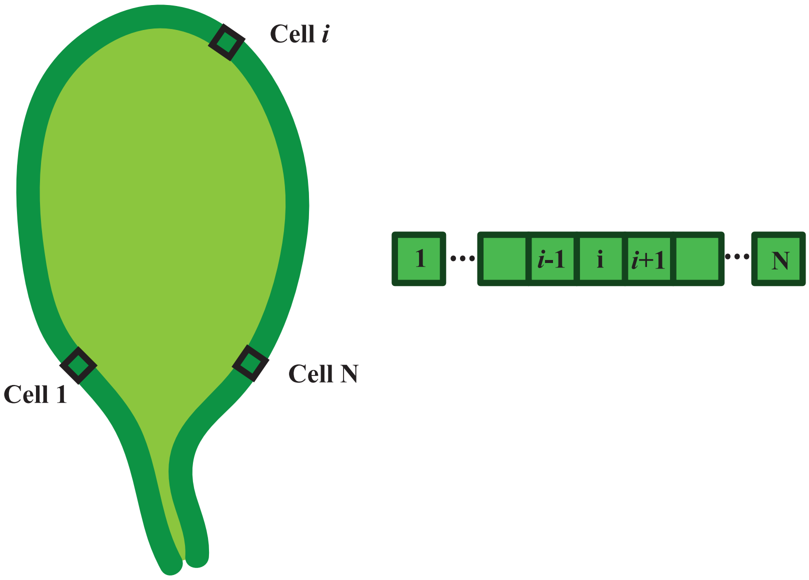

Figure 1: Geometry of the model, showing the positions of the boundary cells on a leaf and the one dimensional domain of square cells.

In the simulation, the time evolutions of the concentrations of auxin, CUC2 and LAX1/2 are described by an ordinary differential equation for each. The rate of change of auxin concentration in cell i has the form:

$$\frac{d}{dt}[AUX]_i = p_1(p_2 - [AUX]_i) - p_3[AUX]_i + \Pi_i + \Delta_i \tag{1}$$

where $[AUX]_i$ is the concentration of auxin in cell i. The first term, $p_1(p_2 - [AUX]_i)$, describes the auxin production in the cell. Auxin levels approach a value of p_2 with a rate controlled by p_1. The second term, $p_3[AUX]_i$, describes

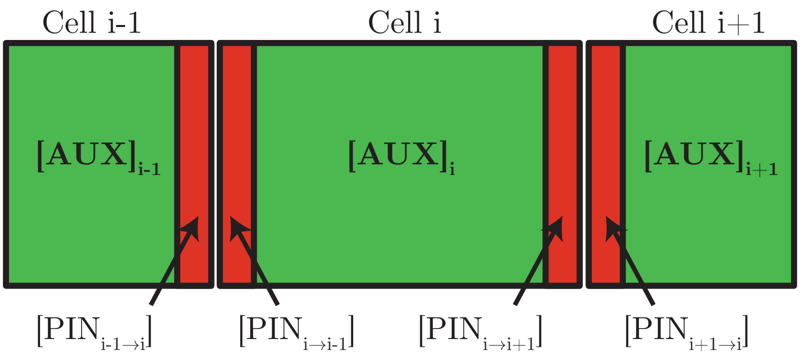

Figure 2: Diagram showing how the PIN concentration on the cell membranes is labeled

the turnover of auxin at a rate of p_3. Π_i represents the PIN1 mediated polar auxin transport into and out of cell i given by:

$$\Pi_i = p_4(-[AUX]_i + [PIN_{i-1\to i}][AUX]_{i-1} + [PIN_{i+1\to i}][AUX]_{i+1}) \qquad (2)$$

The three terms in equation (2) represent, the auxin cell i transports to its neighbours (efflux) and the auxin transported into cell i from the cell's left and right neighbours (influx). p_4 is the auxin transport coefficient and represents the amount of auxin transported per time step.

$[PIN_{i-1\to i}]$ denotes the fraction of PIN1 on the membrane within cell $i-1$ adjoining cell i (Figure 2) and has the form:

$$[PIN_{i-1\to i}] = \begin{cases} \dfrac{6^{[AUX]_i}}{6^{[AUX]_i} + 6^{[AUX]_{i-2}}} & \text{if } [CUC2]_i > \text{Threshold} \\ constant & \text{if } [CUC2]_i < \text{Threshold} \end{cases} \qquad (3)$$

which models the recorded behaviour that PIN1 localises to the membrane neighbouring the cell upstream of the auxin flux [1]. The exponentiation base of six is an arbitrary choice and ensures a strong coupling of PIN1 localisation to walls neighbouring cells of high auxin concentration. CUC2 concentration has an effect on whether PIN1 can preferentially polarise to the cell membrane so equation (3) only holds if CUC2 within the cell in question is above a certain threshold level (in this case, 1). For CUC2 levels below that threshold PIN1 is fixed to the membranes and the pre-existing polarity remains constant [2].

The non polar transport of auxin is modelled by:

$$\Delta_i = p_5 D_i - p_{16} D_i - p_{10}[LAX1]_i D_i - p_{11}[LAX2]_i \quad (4)$$

The four terms represent auxin efflux by diffusion(at a rate, p_5), influx by AUX1 (at a rate, p_{16}), influx by LAX1 (at a rate, p_{10}) and efflux by LAX2 (at a rate, p_{11}) respectively. The D_i term represents non-polar auxin transport:

$$D_i = ([AUX]_{i-1} - 2[AUX]_i + [AUX]_{i+1}) \quad (5)$$

The polar and non-polar transport terms in equations (2) and (4) neglect the fact that transport of auxin from cell to cell is a two stage process, efflux from the cytosol to the extracellular space and influx from the extracellular space to the cytosol of the next cell. Our model combines these two processes and assumes that all auxin that is transported out of the cell is immediately taken up by the next cell. Both the polar and non-polar transport terms (equations (2) and (5)) are mass conserving and when summed up over the range of i are equal to zero.

The concentration of CUC2 in cell i is modelled by the following equation:

$$\frac{d}{dt}[CUC2]_i = \frac{p_6}{1 + p_7[AUX]_i}[CUC2]_i - (p_8 + p_9[AUX]_i)[CUC2]_i \quad (6)$$

Where $[CUC2]_i$ is the concentration of CUC2 in cell i. The first term models the production of CUC2 with a production rate of p_6 and p_7 controls the amount of CUC2 down regulation by auxin. The second term models the CUC2 turnover, with a rate of p_8 and an auxin dependent turnover of p_9.

LAX1 concentrations in cell i are modelled by the following equation:

$$\frac{d}{dt}[LAX1]_i = \frac{p_{12}[AUX]_i^2}{1 + p_{13}[AUX]_i^2}[LAX1]_i - p_{14}[LAX1]_i \quad (7)$$

The form of the first term describes the production of LAX1 and captures the fact that LAX1 and auxin concentration maxima are co-localised but LAX1 maxima form later. p_{12} and p_{13} control the production rate and the maximum levels of LAX1. The second term models a linear turnover of LAX1 at a rate

of p_{14}. LAX1 acts as an auxin importer and the concentration, $[LAX1]_i$, is included in equation (1).

LAX2 concentrations in cell i are modelled by the following equation:

$$\frac{d}{dt}[LAX2]_i = \begin{cases} p_{15}(1 - [LAX2]_i)[AUX]_i & \text{for } \ 26 < i < 74 \\ 0 & \text{otherwise} \end{cases} \tag{8}$$

LAX2 is only present in the interior of the leaf and is also an auxin importer. To model this fact the LAX2 term in equation (1) is an efflux term as auxin is being imported to the interior of the leaf, away from the marginal cells. The concentration of LAX2 is modelled by equation (8). LAX2 is produced at a rate of p_{15} and plateaus at a concentration of 1. LAX2 is only present towards the distal tip of the interior of the leaf. To simulate this observation, LAX2 only has an effect on margin cells that are towards the distal tip of the leaf or, in the case of this simulation, towards the centre of the file of cells. For these simulations the number of cells is 100 and LAX2 only has an affect on cells 26 to 74, the centre half of the leaf margin.

Parameter Values

The various parameters, p_n, in equations (1) to (8) have the physical descriptions depicted in table 1. The values for wild type are listed and the values that were altered to simulate different mutants/treatments are also shown.

The columns have the following meanings:

- WT - Wild type
- L1 - LAX1 mutant
- L2 - LAX2 mutant
- A1 - AUX1 mutant
- TR - LAX1:LAX2:AUX1 mutant
- NPA - Naphthylphthalamic acid treatment (low, medium and high concentration treatments)

Parameter		WT	L1	L2	A1	TR	NPA
p_1	Auxin production rate	0.4					
p_2	Maximum auxin level	10					
p_3	Auxin turnover rate	0.3					
p_4	Auxin transport coefficient	0.4					0.32/0.24/0.16
p_5	Auxin diffusion coefficient	2.5					
p_6	CUC2 production rate	63					
p_7	Sensitivity of CUC2 turnover to auxin	1.7					
p_8	CUC2 turnover rate	3.6					
p_9	Auxin dependent CUC2 turnover rate	0.0018					
p_{10}	Auxin import due to LAX1	1.6	0			0	
p_{11}	Auxin export due to LAX2	2		0		0	
p_{12}	LAX1 production rate	2					
p_{13}	Auxin dependent LAX1 production rate	0.4					
p_{14}	LAX1 turnover rate	1					
p_{15}	Maximun LAX2 level	1					
p_{16}	AUX1 effect on auxin concentration	0.1			0	0	

Table 1: Table of default parameter values for the simulation and the values changed to simulate mutants

Bibliography

[1] R. S. Smith, S. Guyomarc'h, T. Mandel, D. Reinhardt, C. Kuhlemeier, and P. Prusinkiewicz. A plausible model of phyllotaxis. *Proceedings of the National Academy of Sciences of the United States of America*, 103(5):1301–1306, 2006.

[2] G. D Bilsborough, A. Runions, M. Barkoulas, H. W. Jenkins, A. Hasson, C. Galinha, P. Laufs, A. Hay, P. Prusinkiewicz, and M. Tsiantis. Model for the regulation of arabidopsis thaliana leaf margin development. *Proc Natl Acad Sci USA*, 108(8):3424–9, Feb 2011.
