# Supplementary material for: Auxin influx importers modulate serration along the leaf margin
Source: Plant J. 2015 Jul 27;83(4):705–18. doi: 10.1111/tpj.12921 (PMC4949643; doi:10.1111/tpj.12921)
Supplement: Supplementary file 4 [file TPJ-83-705-s004.docx]

**Supporting Information Legends**

**Figure S1.** Mutations in pairs of auxin importers does not lead to a delay in serration. Silhouettes of exemplar leaves at an early stage of leaf development when serration is just initiating in WT primordia (a, b, c), at a mid stage of development when the first serration has clearly formed in WT (d,e,f), and at a later stage of development when multiple serrations have been formed (g,h,i). Leaf shapes are shown for *aux1/lax1* (a,d,g), *aux1/lax2* (b,e,h) and *aux1/lax3* mutant plants (c,f,l). Size bar = a-c 50μm; d-f 200μm; g-i, 500 μm.

**Figure S2.** AUX/PAT/CUC2 model of pattern formation after treatment with NPA. Space/time plot of auxin levels as outputs of an AUX/PAT/CUC2 model in which the leaf margin is depicted as a row of cells (y axis) with the proximal base of the margin at cell 1 and cell 100 and the distal tip of the leaf margin at cell 50. Auxin level is indicated by a spectrum of colour from low (blue) to high (yellow). (a) shows the output after low inhibition of auxin transport by NPA. A pattern is generated after time point 6. (b) shows the output as in (a) but with a moderate inhibition of auxin transport. A weak pattern emerges at approximately time point 11. (c) shows the output as in (a) but with a strong inhibition of auxin transport. Patterning is lost. (d), (e) and (f) show the outputs from a, b, c with an auxin sensitivity window imposed from time point 2, with complete auxin insensitivity being achieved at time point 10. In the case of weak auxin transport inhibition some patterning still occurs at the leaf periphery (b), but in the case of moderate to strong auxin transport inhibition the wave of auxin insensitivity is faster than pattern establishment and no pattern is observed (d,f).

**Model S1.** Description of the mathematical model.
